# Community Contribution to Tuberculosis Care in the Krachi West District of Ghana: A Qualitative Study

**DOI:** 10.1155/2019/5039197

**Published:** 2019-07-14

**Authors:** Samuel Agbenyegah Addy, Eric Osei, Joyce Komesuor, Evelyn Acquah, Prince Justin Anku, Elvis Enowbeyang Tarkang, Farrukh Ishaque Saah, Hubert Amu

**Affiliations:** ^1^Department of Epidemiology and Biostatistics, School of Public Health, University of Health and Allied Sciences, Hohoe, Ghana; ^2^Department of Population and Behavioural Sciences, School of Public Health, University of Health and Allied Sciences, Hohoe, Ghana; ^3^Institute of Health Research, University of Health and Allied Sciences, Ho, Ghana; ^4^Department of Population and Health, University of Cape Coast, Cape Coast, Ghana

## Abstract

**Background:**

Eradicating tuberculosis (TB) is one of the targets of the recently constituted Sustainable Development Goal (SDG) Three. In the light of limitations inherent in prevailing tuberculosis care and the global urgency to improve TB care, decentralising TB care beyond health facilities by harnessing the contribution of communities is essential in ensuring effective tuberculosis care. In this paper, we explored community contribution to TB care in the Krachi West District of Ghana.

**Methods:**

In this qualitative study, 24 TB stakeholders made up of 7 health workers, 9 tuberculosis patients, 4 community health volunteers, 2 treatment supporters, and 2 opinion leaders were interviewed. Data collected were analysed manually, but thematically. Statements of the participants were presented as quotes to substantiate issues discussed.

**Results:**

Community contribution to TB care was low. Most of the community members were not aware of any community level activity towards tuberculosis care. Though patients were mainly the ones responsible for the selection of their treatment supporters, there were instances where health workers selected supporters for them without their consent. Some treatment supporters were also not given any education concerning their roles in supporting their patients, resulting in some patients defaulting treatment and others taking their medications wrongfully.

**Conclusion:**

Our study revealed low community involvement in tuberculosis care in the Krachi West District of Ghana. Community sensitisation on the World Health Organisation's Directly Observed Treatment Strategy (which Ghana adopted in 1994) to increase community involvement in tuberculosis activities is, therefore, recommended.

## 1. Introduction

Tuberculosis remains a major global public health problem as it is the leading cause of death from infectious diseases worldwide [1]. In 2016, about 10.4 million people were infected with TB with an estimated 1,674,000 TB related deaths worldwide [1]. About 25% of these incident cases occurred in the WHO African Region [1].

Despite the proven effectiveness of low-cost strategies to control TB, just one-quarter of all TB patients worldwide receive care in accordance with the international guidelines for diagnosis, treatment, and monitoring [2]. Many of these patients receive inadequate treatment under poorly organized and insufficiently monitored programmes in both public and private sectors while some, in fact, received no treatment at all. This poses a grave danger by encouraging the development of drug-resistant strains, one of the greatest threats to TB control [2].

Ministries of health in many developing countries approach TB control by the provision of curative services through a limited number of specialized institutions located in selected urban centres. This approach was met with only limited success, largely due to the problem of inadequate accessibility [3]. Also, TB case notifications continue to rely heavily on symptomatic individuals voluntarily seeking care at health facilities in many developing countries [4]. As a result, the implementation of the internationally recommended TB control strategy known as the Directly Observed Treatment Short-course (DOTS) requires complementary approaches including community contribution to TB care [5].

DOTS is the main strategy for TB treatment globally and it relies on case detection and treatment with multiple antimicrobial drugs for at least six months. This strategy seeks to integrate the National Tuberculosis Control Program (NTP) with general health services to address the problem of access since it ensures the decentralisation of health care services into the community through a collaborative effort between the community members and health care providers under the community DOTs strategy [6].

The issue of community involvement in public health approaches and in the delivery of health to people is not new [7]. For almost four decades, since the 1978 Declaration of Alma-Ata, community participation in and contribution to health care have been recognized as fundamental for primary health care and accepted as an essential element of many public health interventions. The declaration emphasized “the importance of full and organized community participation and ultimate self-reliance with individuals, families, and communities assuming more responsibility for their own health” which explicitly states that “the people have the right and duty to participate individually and collectively in the planning and implementation of their health care” [8].

The Ghana National Tuberculosis Programme (GNTP) adopted the DOTS strategy in 1994 [9] and had since developed and implemented four strategic plans to address the burden of TB in the country. The general collective efforts have been directed at improving and expanding DOTS to public sector and facilities, expanding private sector participation, and implementing community-based DOTS care [10].

In the Krachi West District, the TB case notification and treatment outcomes have not been encouraging over the years. The district, for instance, notified only 14% of the estimated TB cases in the district 2016 with a treatment success rate of 68% [11] which falls far below the national and WHO recommended treatment success rates.

Understanding the experiences of community contribution to TB care will not only contribute to enhancing the process but will also inform policy direction for better implementation to achieve the programme's targets and goals; hence this current study investigated community contribution to TB care in the Krachi West District of the Volta region, Ghana.

## 2. Conceptual Framework

The conceptual framework of the study is adapted from two theories, Healthcare Utilisation Model (HUM) by Ronald M. Andersen [13] and the Social Ecological Model (SEM) by Urie Bronfenbrenner [14]. The two theories have been adapted because they both have tenets which are very relevant to the study and compatible with the objectives of the study. The Healthcare Utilisation Model, for instance, postulates that people's use of health services is a function of their predisposition to use services, influences which either enable or act as barrier to use, and their need for care [12]. The Social Ecological Model also has five nested, hierarchical levels (individual, interpersonal, community, organizational, and policy/enabling environment) which are influential in the adoption of health decisions of individuals. The role of the community is also highly essential in the adherence to a particular health behaviour by an individual, which in this case is TB [15].

Community contribution to TB care, affected by factors such as occupation of community members, community support system or family support, community resources, accessibility to health facility, and proximity to TB services, is, for instance, captured by the Healthcare Utilisation Model under the tenet of Enabling Factors. Community's understanding of their involvement in TB care and prevention is also posited in the Healthcare Utilization Model. Also, community's awareness of complications that can arise from defaulting TB treatment, the potential of the TB patient spreading the disease to other members in the community, and the cost and burden that can emanate from multidrug resistance TB can greatly influence their contribution and involvement in TB care, management, and control activities.

Both the HUM and SEM recognise community characteristics and relationships among community leaders and other institutions that can influence their contribution to TB care and control. A clear understanding of the benefits of community contribution to TB care will, therefore, increase their involvement in TB activities.

This study also sought to explore communities' contribution to TB care, training of treatment supporters to increase their knowledge on TB and barriers to TB care. These barriers are classified into three groups by the HUM as follows: sociodemographic (predisposing) factors, health service-related (enabling) factors, and sociologic (reinforcing) factors. These factors shape the roles communities play in the control and prevention of TB such as awareness creation during durbars, addressing stigmatization, facilitating the implementation of Directly Observed Treatment [DOTs] strategy, choosing of community volunteers, community education, and involvement, and supporting patients in some of their needs, including provision of DOT, food, and housing.

**Figure 1:**
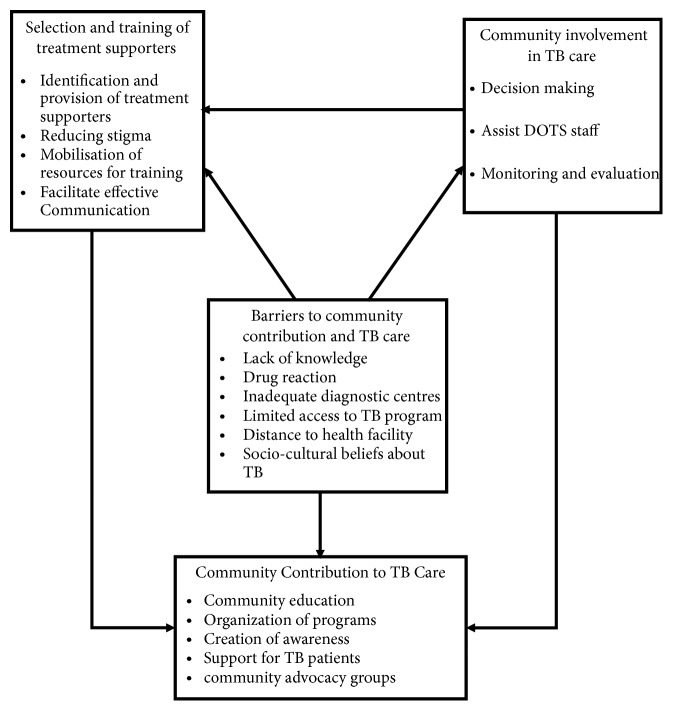
*Conceptual framework*. Source: Adapted from Andersen and Newman [12] and Peterson (2016).

Two possible outcomes of community contribution to TB care are shown in the framework (Figure 1). Firstly, community involvement in TB activities will lead to better understanding of TB and consequently reduce stigma attached to the disease. Also, this will in turn lead to community members availing themselves to be used as treatment supporters. With this the community will be able to contribute to TB activities. On the other hand, barriers such as lack of knowledge, limited access to TB program, and distance can affect either the community contribution or their involvement in TB activities.

## 3. Materials and Methods

### 3.1. Setting

The study was conducted in the Krachi West District of the Volta Region of Ghana. The total population of the district was 57,308 [11]. The district is made up of about 350 communities, of which more than 30 percent are scattered on the lake and along its banks, only accessible by the lake. Regarding health facilities, there is only one hospital serving the entire district, three (3) health centres, nine (9) Community-Based Health Planning Services (CHPS), and one Reproductive and Child Health clinic (RCH).

The average household size in the district is 4.3 persons per household. Almost half (44%) of households with a size of three members have only one sleeping room. This can facilitate the spread of TB in the area [16]. The district has an average expected TB cases per year of around 90. However, only about 25% of these cases are detected annually. TB cases receive free treatment once diagnosed in the district as every part of the country and is expected to implement the community DOTS strategy.

### 3.2. Study Design and Population

This study adopted a qualitative approach using an explanatory design [17]. The study population for this study was made up of registered TB patients in the district from December 2015 up to the time the data were collected, DOTs centre in charge, district pharmacist, laboratory technician in charge of sputum examination, the district TB coordinator, treatment supporters, community health volunteers, and opinion leaders.

### 3.3. Procedures

Participants were selected using a purposive sampling technique. This technique was chosen in order to reach persons who have experienced the phenomenon of this study, thus TB treatment, care, and control. The study purposively selected nine (9) TB patients, seven (7) health workers, four (4) community volunteers, two (2) TB treatment supporters, and two (2) opinion leaders by saturation.

With the support of three research assistants, the researchers collected data from the participants through in-depth interviews. Data were collected using interview guides self-developed for each group of participants. Appointments were booked with all participants in order to ensure that the interviews took place at their own chosen convenient times and sites. Interviews were conducted in either English (for all health professionals), Ewe, or Twi (for most TB patients, treatment supporters, and community members). Quality was ensured through joint assessment of the sampling approach and ongoing review of transcripts to explore areas for further probing.

### 3.4. Data Analysis

Audio recordings of in-depth interviews were transcribed together with the field notes. Interviews conducted in Ewe and Twi were also transcribed into English. Transcriptions were proofred and edited before it was thoroughly read and manually analysed thematically. Codes were generated and joined to create themes based on the specific objectives of the study and other emerging themes from the data. Quotes from participants were presented to substantiate issues discussed while a frequency table was used to present the sociodemographic characteristics of the participants.

Community contribution to TB care was operationalized as any conscientious efforts through organized activities initiated and based in the community with the aim of aiding the prevention and management of TB (this is a more active position of the community).

Community involvement though forming part of community contribution was defined for the purpose of this study as a passive role of community individuals towards impacting the management process for TB treatment.

### 3.5. Ethical Approval

The study obtained ethical approval from the Dodowa Health Research Centre Institutional Review Board, Ghana* (Reference number: DHRCIRB/15/05/17)* before the study was conducted. Permission was taken from the Krachi West District Health Management Team administration and community leaders (including chiefs and assembly members).

## 4. Results

### 4.1. Sociodemographic Characteristics of Participants

The study involved TB patients (37.5%), health workers (29.1%), community volunteers (16.7%), TB treatment supporters (8.3%), and opinion leaders (8.3%). Regarding age, 33.3 percent were in their 40s while a few (4.2%) were in their teens and about a fifth (20.9%) were aged 60 years and more. About 75 percent were married, while a fourth (25%) had never been married. Most of the study participants (79.2%) were Christians, whereas Muslims and African traditionalists constituted 12.5 percent and 8.3 percent, respectively. Half of the study participants (50%) were Guans and more than a third (37.4%) were Ewes. The highest level of education of participants ranged from no formal education to tertiary. A comparative majority (41.7%) had had tertiary education while more than a fourth (29.2%) had had JHS/JSS/Middle school education. Similar number of participants (8.2%) had had primary and SHS/SSS/A'level/O'level education (Table 1).

### 4.2. Community Contribution to TB Care

The study sought to identify contributions of communities to TB care in the Krachi West District. Participants were to proffer ways in which communities contribute to TB care. Two themes evolved, no community contribution to TB care and low community contribution to TB care. Most of the study participants noted that there was low community contribution towards TB care. They explained that there are no organized and established activities being done by the community and their members aimed at contributing to TB care. The following quotes summarise their views.

Okay, as for me, I don't see anything like that in this community, I am not sure there is. But as far as I know we don't do anything here towards TB on our own but there is this doctor who lives in Kumasi, but he is actually from this village, every year he has been coming here to organise health screening for us but not on TB anyway.

–Assembly Member, Male, 51 years.

The community members themselves don't do anything concerning TB, however, we the health workers as part of our routine home visits do talk to them concerning certain diseases but not on TB specifically even though we include it at times.

–Health worker, Female, 30 years.

However, they acknowledged that the community together with health workers and volunteers organize health durbars which sometimes provide education on TB and other diseases. They mentioned health talks during community durbars, stigma reduction programmes, and patients support as communities' contributions to TB care. Their views are as shown below.

During durbars, they (community elders) used to invite health workers to come and educate us on how some communicable diseases can be contracted. Like if you smoke cigarette, if you drink alcohol, if you live in an overcrowded environment, it is not good since you are at risk of getting so many diseases including TB. And then Also, if you know you have the disease (TB), if you are coughing, you have to cover your mouth with handkerchief, if not, if someone is close to you, the person can contract the disease.

– TB patient, Male, 46 years.

What happens is that most relatives do not like to get close to the TB patients because they think the patient has HIV/AIDS. We make the people understand through our health education talks that, the disease is not contracted by touching the patient, but transmitted through the air especially through cough droplets and once the person is on treatment or covers the mouth before coughing, he/she may not be able to spread the disease.

– Community volunteer, Male, 37 years.

In my case, from the time I was put on treatment, some of my friends have been supporting me. Anytime they (friends) visited me, they do give me money. Like 5 or 10 cedis especially those I do the fish business with. Some also have been fetching fire wood for me as well as fetch water.

-TB Patient, Female, 65 years.

What I know is we have TB coordinators who are directly in charge of cases, so when there are cases it involves the lab confirm it then we initiate treatment. We locate caretakers in the community to make sure that the patient regularly follows the treatment schedule and the follow up are also done.

– Health worker, Male, 24 years.

### 4.3. Selection and Training of Treatment Supporters

We also sought to determine the involvement of TB patients in the selection of their treatment supporters and training of treatment supporters. The two major themes obtained were selection of treatment supporter together with patients and not having treatment supporter at all. For those who had treatment supporters, it was explained that most of the patients were involved in the selection of their treatment supporters. Nevertheless, some treatment supporters were not given proper couching concerning the supervision of the patients. The following quotes summarise their views.

So, what happen is that once the person is diagnosed and he/she is having a relative, we ask if the relative can actually see to it that the he/she actually take the drugs and can encourage the patient to take the drugs more frequently. We also looked at who the supporter is to the patient, we don't just choose any body. And if the patient is older than the treatment supporter, a lot of things are compromised because the father's authority or parent's authority is at play here, that is one thing we look out for, however, if the patient's resident is close to a health facility, and there is no supporter as such, then we assign a CHN or an enrolled nurse in that community to ensure that the patient takes the medicine for successful treatment.

– Health worker, Male, 31 years.

My daughter, was the one who sent me to the hospital so she was told that when we come back to the house, every morning she should remove the drug for me and make sure I take them after which she should tick a column in a small yellow card that they gave us. They (health workers) also asked me if I will be comfortable with my daughter supervising my treatment and I told them yes.

– TB patient, Female, 43 years.

Me, the man (the doctor) called me and he told me what the problem was, and he added that, so this man (a male nurse) will be the one who will be giving me the drugs each time I am to take the drug. I don't know the man (nurse). But the man only comes here once in a while to make sure that I take the drug.

– TB patient, Male, 51 years.

As for treatment supporter, I don't have one but one guy came here last month to see how far, and the person who gave me the drugs too, is in the hospital there but as for the drugs I take them myself. They gave me a card to be ticking each time I take the drug, so that was what I have been doing.

– TB patient, Female, 47 years.

We have not train them (treatment supporters) as in a formal training before, but what we do is that, we ask the patient to bring the supporter and then we educate the supporter on how he or she will go about supporting the patient and then we tell the supporter how he or she will go about supporting the patient and then we encourage him or her to be able to effectively support the patient.

– Health worker, Male, 31 years.

### 4.4. Barriers to Community Contribution to TB Care

We investigated barriers to communities' contribution to TB care. Two major themes regarding barriers to communities' contribution to TB care were identified, namely, stigmatisation and inadequate knowledge of community members. Regarding stigmatization, participants explained that due to the stigma attached TB, though individuals are willing to involve themselves in the management, they are afraid to be stigmatized or receive negative reaction from other community members. Few of such views proffered by the participants on stigmatization include the following.

So like when we give them (community members) the information, then we ask them to report anybody with a cough to the facility but you see it is impolite according to our cultures to tell the person to go to the hospital, the person will think that, you are stigmatizing as a result until the person report to the health facility before we can follow up and then take the necessary actions.

– Health worker, Male, 31 years.

Like they usually combine TB program with HIV so when you ask for a community durbar, because you are combining the two, that is my observation, because you are combining the two, people won't come, the community will always think (asi me si) belongs to that club or whatever, so they won't even come, so such durbars are difficult to organise here.

– Community volunteer, Male, 41 years.

The way I have been suffering from these people (community members). It is the sickness that made me turn old lady all of a sudden and because of the way I have grown lean, most of them stopped coming to my house. As a result, they do not contribute in any way to support me get well. There was a time one of them came here to tell me that, she had a dream, and I was dead. So, I don't go anywhere. Always, this is where I sleep alone.

– TB patient, Female, 65 years.

Also, in relation to community members not having adequate knowledge about TB care, it was explained that though the community and individuals are willing to involve themselves and contribute to management, they have inadequate knowledge on what they can do to support. They also do not understand what ways to contribute to the management process. For instance, a 31-year opinion leader noted,* “most of us (community members) do not know much about TB. We would like to help and be more supportive but we don't know how we can be more helpful since no nurse or doctor came to tell us about it (how to support patients).”* Similarly, a 40-year old community volunteer said, “*The community lacks understanding of the disease and where and when to seek care. Even relatives don't know how to prevent themselves from getting the infection while still helping the patient. As a result, they all desert him/her.”*

## 5. Discussion

This study explored community contribution to tuberculosis care in the Krachi West District of Ghana. It was found that there was little to no community contribution to TB care in the district by community members. Most TB patients were involved in the selection of their treatment supports. However, some treatment supporters were not given proper training regarding how the medications should be taken and supervision of patients done. This resulted in some of the patients defaulting treatment. The major barriers which hindered community contribution to TB care were stigmatization and lack of knowledge on TB among the community members.

The low level of community contribution to TB care realised in the present study comes at the backdrop that according to the WHO [7], the main role of communities in the fight against TB is for them to contribute to detection of new TB cases and to improve adherence to treatment. Community volunteers and people who have ever had TB according to the WHO [7] are expected to engage in referring people with chronic cough for sputum examination, sharing experiences and serving as advocates during community outreach, promoting DOTS services, serving as treatment partners for people with TB, assisting staff at health centers, and giving talks in schools and sharing information about the disease to help reduce stigma and help people to identify TB symptoms. Others include storing drugs for people with TB, reminding people with TB about follow-up visits, accompanying people with TB to nearby health facilities, and referring people with TB who experience adverse reactions to drugs [18]. These activities were, however, largely missing.

Regarding the findings concerning community activities towards TB care, the results were in line with that of Amenuvegbe, Anto, and Binka [19] which reported that community-based TB activities are activities that are conducted outside the premises of formal health facilities, for example, activities conducted within community-based structures such as schools, places of worship, and households. Hadley and Maher [20] also identified that community contribution to tuberculosis care is any community-based TB activity that facilitates the implementation of the community DOTS strategy. They further argued that community education, involvement, and organization around TB issues can encourage a feeling of community ownership of TB programs and reduce stigma. They also emphasised that communities can help address the patient's interim needs including the provision of DOT, food, and/or housing. However, the reasons for the unawareness may be that even if these activities are being carried out, there is low involvement from the members since majority of the community members were not aware of the existence of these activities as mentioned by few of the participants. In the conceptual framework, Andersen and Newman [12] identified income level, community resources, accessibility, waiting time at the hospital, and proximity to help as factors which can determine community participation in health activities.

Concerning patient's involvement in the selection of treatment supporters, the findings support that of Olukolade et al. [21], who identified in their study that patients are involved in the selection of treatment supporters which is essential for treatment success. The reason could be that when patients are left on their own, they may not be committed to taking their drugs according to the treatment regimen. The treatment supporters can encourage, persuade, and remind the patient of the unwholesome consequences for missing their drugs. This support could enable patients to adhere to the treatment regimen. Supporting a patient at times involves collecting the drugs on behalf of the patient when he/she is not able to make it to the DOTS Centre. This finding is consistent with the WHO [18] recommendation of health providers helping the TB patient to choose a treatment supporter.

In relation to training of treatment supporters, the present study has showed that even though most of the treatment supporters were trained on how the patients should take the drugs as well as their roles as treatment supporters, that is, to ensure that the patient complete the treatment successfully, some treatment supporters were not properly couched leading to treatment default [18]. The implementation of the DOTs strategy requires that the patient identify someone in the community who is close to him/her to ensure that he/she takes the drugs correctly as prescribed. Also, the patient should be involved in the selection of the treatment supporter. However, the findings of the current study show that few patients were given treatment supporters without their involvement. Also, most of the treatment supporters were not trained on how the patient should take the drug, a finding that is inconsistent with WHO [18] recommendation as stated earlier. In relation to the conceptual framework, the role of treatment supporters is very essential for successful completion of treatment. If treatment supporters are not trained well, treatment success will be compromised.

Concerning stigmatization as a barrier to community contribution to TB care and taking views from health workers and some TB patients on their experiences in the execution of duties relating to TB services and experiences whilst on treatment, it can safely be asserted that even health workers are stigmatizing against people with TB as well as some community members. This finding is congruent to the findings by Gebremariam, Bjune and Frich [22], and Reyes and Amores [23] conducted in Ethiopia and Philippines, respectively. The authors argued, respectively, that stigma associated with TB coupled with a lack of adequate communication with health professionals is prevalent in countries with high HIV cases, where HIV and TB coinfection serves as a common barrier to TB care. Stigma is a barrier that presents a serious obstacle to successful TB control. Health-seeking behaviour is made up of a balance of costs and benefits to the patient. The benefits of getting well are unlikely to outweigh the costs of social and family rejection and loss of employment and accommodation at the early stages of the disease. A direct approach to address stigma in a society may involve understanding and addressing the beliefs and attitudes of the community towards the disease. However, if authorities did not put conscious efforts in place to address this situation, people living with the disease may not likely seek care.

Regarding the present study's finding that lack of knowledge about TB care hinders contributing to TB care, this finding supports the argument of Waisbord [24] in which the author argued that lack of knowledge of TB care such as length of treatment and prevention affects the care and control of the disease. Also, this is in congruence to the conceptual framework in which Andersen and Newman [12] posit that knowledge influences communities' activities and influence on health behaviour such as the care and control of TB.

Despite the important findings made in the present study, the potential limitations of the study are worth mentioning. As the study relied on the verbal reports of TB patients, treatment supporters, health professionals, and community leaders regarding the state of TB care in the district, the results realised could have suffered from recall bias, as some of the participants might not have remembered issues which occurred in the past vividly. Although the study ensured that the interviews were conducted among various stakeholders in the management of TB, the fact that participants were selected purposively might have subjected the study to selection and response biases. The various groups being involved in the study were, however, to ensure that the responses provided by the participants in one stakeholder group are verified or otherwise by others in other groups.

## 6. Conclusion

Community contribution was low in the present study. The implication of this finding is that the operational effectiveness of the DOTs strategy in eliminating TB will be compromised. In effect, Ghana may not be able to achieve the WHO's community TB DOTs strategy recommendation of case notification of more than 70% and treatment success rate of 85% which all aim at ending TB by the year 2030. The study, therefore, underscores the need to establish effective communication between community TB treatment supporters and the health units. All possible means of harnessing the community contribution to TB care are recommended in order to further increase TB notification and treatment success rates.

## Figures and Tables

**Table 1:** Sociodemographic characteristics of participants.

Variable	Frequency	Percentage (%)
*Status of participant*		
Health Workers	7	29.2
TB patients	9	37.5
Community volunteers	4	16.7
TB treatment supporters	2	8.3
Opinion leaders	2	8.3
*Age*		
15-19	1	4.2
20-29	5	20.8
30-39	3	12.5
40-49	8	33.3
50-59	2	8.3
60+	5	20.9
*Sex*		
Male	15	62.5
Female	9	37.5
*Marital status*		
Married	18	75
Never married	6	25
*Religion*		
Christianity	19	79.2
Islam	3	12.5
African Traditional Religion	2	8.3
*Ethnicity*		
Guan	12	50
Ewe	9	37.4
Mole-Dagbani	3	12.6
*Highest Level of Education*		
Primary	2	8.3
JHS/ Middle school	7	29.2
SHS/SSS/O', A' level	2	8.3
Tertiary	10	41.7
Non-formal	3	12.5

## Data Availability

Data will be made available upon request.

## References

[1] World Health Organisation (WHO) (2017). *Global Tuberculosis Report*.

[2] Danso E., Addo I. Y., Ampomah I. G. (2015). Patients compliance with tuberculosis medication in Ghana: evidence from a periurban community. *Advances in Public Health*.

[3] Fiseha D., Demissie M. (2015). Assessment of directly observed therapy (DOT) following tuberculosis regimen change in Addis Ababa, Ethiopia: a qualitative study. *BMC Infectious Diseases*.

[4] Lorent N., Choun K., Thai S. (2014). Community-based active tuberculosis case finding in poor urban settlements of Phnom Penh, Cambodia: a feasible and effective strategy. *PLoS ONE*.

[5] Tilahun G., Gebre-Selassie S. (2016). Treatment outcomes of childhood tuberculosis in Addis Ababa: a five-year retrospective analysis. *BMC Public Health*.

[6] Datiko D. G., Yassin M. A., Tulloch O. (2015). Exploring providers' perspectives of a community-based TB approach in Southern Ethiopia: implication for community-based approaches. *BMC Health Services Research*.

[7] World Health Organization (WHO) (2008). *Community Involvement in Tuberculosis Care and Prevention: Towards Partnerships for Health*.

[8] World Health Organization (WHO) (2005). *The Bangkok Charter for Health Promotion in a Globalized World*.

[9] Pehr J. L. (2010). Health care and infrastructure in Accra, Ghana. *Advanced Issues in Urban Planning*.

[10] Bonsu F. A., Hanson-Nortey N. N., Afutu F. K. (2014). *The National Tuberculosis Health Sector Strategic Plan for Ghana 2015–2020*.

[11] Krachi West District Health (KWHD) (2016). Krachi west health directorate annual report.

[12] Andersen R., Newman J. F. (2005). Societal and individual determinants of medical care utilization in the United States. *Milbank Quarterly*.

[13] Andersen R. M. (1968). *Families' Use of Health Services: A Behavioural Model of Predisposing, Enabling and Need Components*.

[14] Bronfenbrenner U. (1977). Toward an experimental ecology of human development. *American Psychologist*.

[15] Centers for Disease Control and Prevention (CDC) (2015). *The Social-Ecological Model: A Framework for Prevention*.

[16] Ghana Statistical Service (2014). *2010 Population and Housing Census: National Analytic Report*.

[17] Smith A. M. (2012). Research methodology: a step-by-step guide for beginners. *Nurse Education in Practice*.

[18] World Health Organization (WHO) (2010). *Management of Tuberculosis Training for Health Facility Staff*.

[19] Amenuvegbe G. K., Francis A., Fred B. (2016). Low tuberculosis case detection: a community and health facility based study of contributory factors in the Nkwanta South district of Ghana. *BMC Research Notes*.

[20] Hadley M., Maher D. (2000). Community involvement in tuberculosis control: lessons from other health care programmes. *The International Journal of Tuberculosis and Lung Disease*.

[21] Olukolade R., Hassan A., Ogbuji Q. (2017). Role of treatment supporters beyond monitoring daily drug intake for TB-patients: findings from a qualitative study in Nigeria. *Journal of Public Health and Epidemiology*.

[22] Gebremariam M. K., Bjune G. A., Frich J. C. (2010). Barriers and facilitators of adherence to TB treatment in patients on concomitant TB and HIV treatment: a qualitative study. *BMC Public Health*.

[23] Reyes K., Amores J. C. (2014). *Barriers of Early TB Diagnosis among The Poor in Highly Urbanized Areas in The Philippines (No. 2014-18)*.

[24] Waisbord S. (2004). *Behavioral Barriers in Tuberculosis Control: A Literature Review*.

